# Association of humidity and precipitation with asthma: a systematic review and meta-analysis

**DOI:** 10.3389/falgy.2024.1483430

**Published:** 2024-12-06

**Authors:** Wangyang Gu, Dan Xie, Qinpeng Li, Huike Feng, Yihao Xue, Yang Chen, Jingyi Tang, Yushi Zhou, Dan Wang, Shilu Tong, Shijian Liu

**Affiliations:** ^1^ Department of Big Data Center, Sanya Women and Children's Hospital Affiliated to Hainan Medical University, Sanya, China; ^2^ Hainan Branch, Shanghai Children’s Medical Center, School of Medicine, Shanghai Jiao Tong University, Sanya, China; ^3^ Department of Epidemiology and Statistics, School of Public Health, School of Medicine, Shanghai Jiao Tong University, Shanghai, China; ^4^ Department of Epidemiology and Statistics, School of Public Health and Social Work, Queensland University of Technology, Brisbane, QLD, Australia; ^5^ Chinese Centers for Disease Control and Prevention, National Institute of Environmental Health, Beijing, China; ^6^ Shanghai Children’s Medical Center, School of Medicine, Shanghai Jiao Tong University, Sanya, China

**Keywords:** humidity, precipitation, asthma, systematic review, meta-analysis

## Abstract

**Introduction:**

The relationship of asthma with humidity and precipitation remains controversial. The objective of this study was to investigate the association of increased humidity and precipitation with asthma risk.

**Methods:**

A comprehensive systematic search was conducted across various databases, including PubMed, Embase, Cochrane Library, Web of Science, Chinese Wanfang, CQVIP, and CNKI. A total of 21 studies with 1,052,960 participants from 9 countries or regions were included. The fixed and random effect model were used to analyze the data.

**Results:**

The study revealed a pooled odds ratio (OR) of 1.0489 [95% confidence interval (CI): 1.0061, 1.0935] for the association between humidity and asthma risk. Specifically, among individuals under 18 years of age, the OR (95% CI) was 1.0898 (1.0290, 1.1541). Furthermore, the OR (95% CI) for developing countries or regions was 1.0927 (1.0220, 1.1684), while it was 1.1298 (0.9502, 1.3433) for regions with a high latitude (41°–51°). The pooled OR for precipitation and asthma risk was 0.9991 (0.9987, 0.9995). The OR (95%CI) values were 0.9991 (0.9987, 0.9995), 0.9991 (0.9987, 0.9995) and 0.9990 (0.9986, 0.9994) in people above the age of 18, developing countries or regions, and middle latitudes (31°–41°), respectively.

**Discussion:**

The impact of humidity on asthma risk is particularly pronounced among individuals below 18 years of age, people living in developing countries or regions and in regions located in high latitudes. And the influence of precipitation on asthma persons over the age of 18, developing countries or regions, and middle latitudes significantly. Increased humidity appears to elevate asthma risk, and increased precipitation may reduce the risk. In addition, there appears to be a combined effect of humidity and precipitation on asthma.

**Systematic Review Registration:**

PROSPERO, identifier, CRD42023482446.

## 1 Introduction

Asthma is a chronic ailment that has a substantial impact on human health and well-being (1). Its primary manifestations consist of airway constriction, lung inflammation, excessive mucus production, and respiratory distress (2, 3). This condition can affect individuals across all age groups, with a prevalence ranging from 5 to 10% worldwide (4). It is worth noting that approximately 3.6% of adults with asthma suffer from severe refractory asthma (5), while around 7.5% of children globally are afflicted by asthma (6). According to the report of World Health Organization (WHO), a global estimate of 262 million individuals were diagnosed with asthma in 2019, leading to approximately 455,000 deaths. Although the causes of asthma remain largely unknown, some evidence suggests that environmental factors, such as climatic changes, play a role in exacerbating asthma symptoms.

The association between global climate change and respiratory health has attracted significant public interests. Among the many climatic factors, three meteorological factors including temperature, humidity and precipitation have been extensively studied (7–10). Temperature, in particular, has been linked to various negative health outcomes, such as heightened cardiovascular and respiratory morbidity and mortality (11, 12). However, investigations into the effects of ambient humidity and precipitation on asthma risk have produced conflicting findings, thereby contributing to the ongoing debate. For example, a study found that asthma patients aged over 14 years had a 1.3-fold higher relative risk of doctor visits when exposed to 95% humidity compared to lower levels (13). Conversely, a study by Hu and his colleagues indicated that excessively low humidity may be a risk factor for severe respiratory symptoms in children with asthma (14). Additionally, the Strauss investigation demonstrated that increasing the moisture of inhaled air at room temperature effectively reduced bronchospasm response to exercise in individuals with asthma (15). Schinasi found that heavy precipitation events in summer may lead to worsening of asthma, but the underlying mechanisms remain unclear (16). In addition, studies have shown that low humidity, increase the content of particulate matter, thus increasing the risk of death and disease (17). Precipitation was negatively correlated with asthma (18). Areas with low humidity was associated with higher rates of childhood asthma hospitalizations (19). Other studies have found that heavy precipitation and humidity jointly affect asthma. For example, areas with wetter weather have increased risk of indoor mould and fungal spores, leading to increased risk of asthma (17). Heavy rainfall and flood can lead to residential damp and mold proliferation, thus affecting indoor air quality and asthma symptoms (20).

Despite the increasing recognition of the influence of climate change on respiratory health, the association between humidity and precipitation and asthma is still inconsistent. Additional research is necessary to reconcile the divergent outcomes and elucidate the exact mechanisms that underlie this connection.

To investigate the influence of environmental humidity fluctuations and precipitation on the risk of asthma, a systematic review and meta-analysis was conducted. Furthermore, this review sought to explore the modification effects by varying exposure factors, such as age, regional economic status, and latitude on the link between humidity and precipitation and the risk of asthma.

## 2 Methods

According to previous research, the relative humidity was used as a measurement of humidity (8). For the purpose of review, humidity change was defined as a change in humidity levels compared to lower or moderate levels observed over a specific period within the same geographical area (13). Precipitation change was then defined as a significant increase in precipitation relative to low or moderate precipitation levels in the same period and region. Asthma risk was defined as a change in the incidence of asthma in an area over a period of time or the number of medical visits for asthma attacks in the asthma population in that area over a certain period of time (16, 21, 22), with data derived from either self-reporting or medical records obtained from hospitals or other relevant databases (23).

This study was conducted following the guidelines of the Priority Reporting Items for Systematic Evaluation and Meta-Analysis (PRISMA). Additionally, it was registered through PROSPERO (CRD42023482446, https://www.crd.york.ac.uk/).

### 2.1 Search strategy

A comprehensive literature research was performed to identify pertinent studies through the utilization of multiple databases, namely PubMed, Embase, Cochrane Library, Web of Science, Wanfang, China Science and Technology Journal Database (CQVIP), and China National Knowledge Infrastructure (CNKI). The search encompassed studies published until April 7, 2024, without any limitations on language and encompassed diverse study designs to ensure inclusiveness. And search strategy used the following terms: ((Humidity OR rainfall OR precipitation) OR temperature OR (“atmospheric pressure” OR “air pressure” OR “barometric pressure”) OR (climate OR Meteorolog* OR weather) OR (“wind speed” OR “wind velocity”) OR “sunshine duration”) AND (asthma OR “asthma incidence” OR wheeze). Additionally, the references cited in the included studies were retrieved to identify any relevant articles.

### 2.2 Inclusion and exclusion criteria

To guarantee the incorporation of high-quality and pertinent studies in the review, we formulated specific inclusion criteria. The criteria encompassed epidemiological investigations employing diverse study designs, including cross-sectional, case-control, cohort, ecological, time-series, and case-crossover methodologies. The selected articles were required to concentrate on the relationship between asthma and meteorological variations, with a particular emphasis on humidity and precipitation. The diagnosis of asthma was determined in accordance with the International Classification of Diseases or relevant national and local guidelines. The assessment of various outcomes related to asthma, such as hospitalizations, emergency department visits, outpatient visits, asthma mortality, asthma symptoms, and asthma diagnosis, should be included. The studies should investigate the association of asthma risk with humidity and precipitation, presenting odds ratios (ORs), relative risks (RRs), hazard ratios (HR), or beta coefficients for the incidence of asthma, accompanied by corresponding 95% confidence intervals (CIs) or data that can be used to estimate these outcomes. Additionally, the study should include a minimum sample size of 50 cases, as suggested by Cong et al. (24).

To ensure the quality and relevance of the studies incorporated into our analysis, we implemented stringent exclusion criteria. These criteria led to the exclusion of duplicate reports, studies with incomplete data, case studies, editorials, and conference proceedings. In instances where multiple reports were available, only the most comprehensive and recent version was selected for inclusion. Reviews, conference abstracts, dissertations, audits, policy analyses, book reviews, pilot studies, opinion pieces, and studies in the planning stage (unless listed in a study catalogue) were also excluded. Furthermore, studies that did not specify sample sizes or included fewer than 50 participants were also excluded.

### 2.3 Literature screening and data extraction

The studies underwent a stringent screening procedure to eliminate any instances of duplicate publications. Initially, titles and abstracts were reviewed for relevance, followed by a detailed examination of the full texts to confirm compliance with the inclusion criteria and to exclude any literature meeting the exclusion criteria.

The essential components of the studies included in the analysis were systematically gathered and recorded, encompassing vital details such as the primary author's identity, publication year, study location, research design, demographic characteristics of participants (gender and age), sample size, meteorological data sources, asthma outcome measures, and the corresponding effect estimates (OR, RR, HR, or beta coefficients) with their respective 95% confidence intervals, pertaining to the relationship between humidity, precipitation, and asthma risk. Furthermore, information regarding the confounding factors was also extracted, ensuring a comprehensive evaluation of the studies included.

### 2.4 Quality assessment

The quality assessment tool used in the time-series and case-crossover studies in this review were adapted from prior studies (25–27). The tool encompassed several key items, such as asthma diagnosis validation, exposure measurement quality for humidity or precipitation, and confounder adjustment. Each item was scored from 0 to 3 based on specific evaluation criteria. Regarding the validation of asthma diagnosis, a score of 1 was assigned if confirmed by International Classification of Diseases (ICD) coding or valid criteria; otherwise, 0. For exposure measurement quality, a score of 1 was assigned if measurement frequency was at least once a day and missing data <25%; otherwise, 0 (27). In terms of adjusting for potential confounders, a score of 1–3 was given if the analysis accounted for long-term trends, seasonal variations, and temperature; otherwise, 0. For quality assessment of ecological studies, an adapted version of the Newcastle-Ottawa assessment scale (NOS) was used (28). For cross-sectional studies, the Agency for Healthcare Research and Quality (AHRQ) 11-item checklist was used, with scores of 1 for “Yes” and 0 for “No” or “Unclear”. Quality levels were categorized as low (0–3), moderate (4–7), and high (8–11) (29). This comprehensive tool enabled assessment of methodological strength and potential quality impact on findings.

### 2.5 Statistical analysis

The study used various effect size indicators, including ORs, RRs, HRs, and *β* coefficients, along with their 95% CIs, to report the association of humidity or precipitation exposure with asthma risk. Adjusted models were employed to compare the risk estimates of the highest exposed group with the lowest (or reference group). For comparability, RRs and HRs were converted to ORs and then combined (30). The combined risk estimates were visually represented in a forest plot. The heterogeneity among the studies was assessed using the *I^2^* statistic and *p*-values. If there was a significant heterogeneity (*I^2^* > 50% or *p* < 0.1), a random effects model was applied; otherwise, a fixed effects model was chosen. Sensitivity analyses, excluding one study at a time, were conducted to assess the robustness of the results. Publication bias was assessed using Egger's test. Subgroup analyses were performed based on age, regional development level, and latitude. Age was divided into two groups: <18 and ≥18 years. Regional development was classified into developing and developed categories. Latitude was partitioned into three zones: low (21°–31°), middle (31°–41°), and high (41°–51°). All statistical analyses were carried out using R 4.3.0. A two tailed *p*-value <0.05 was considered statistically significant.

## 3 Results

### 3.1 Literature screening and characteristics of included studies

**Figure 1:**
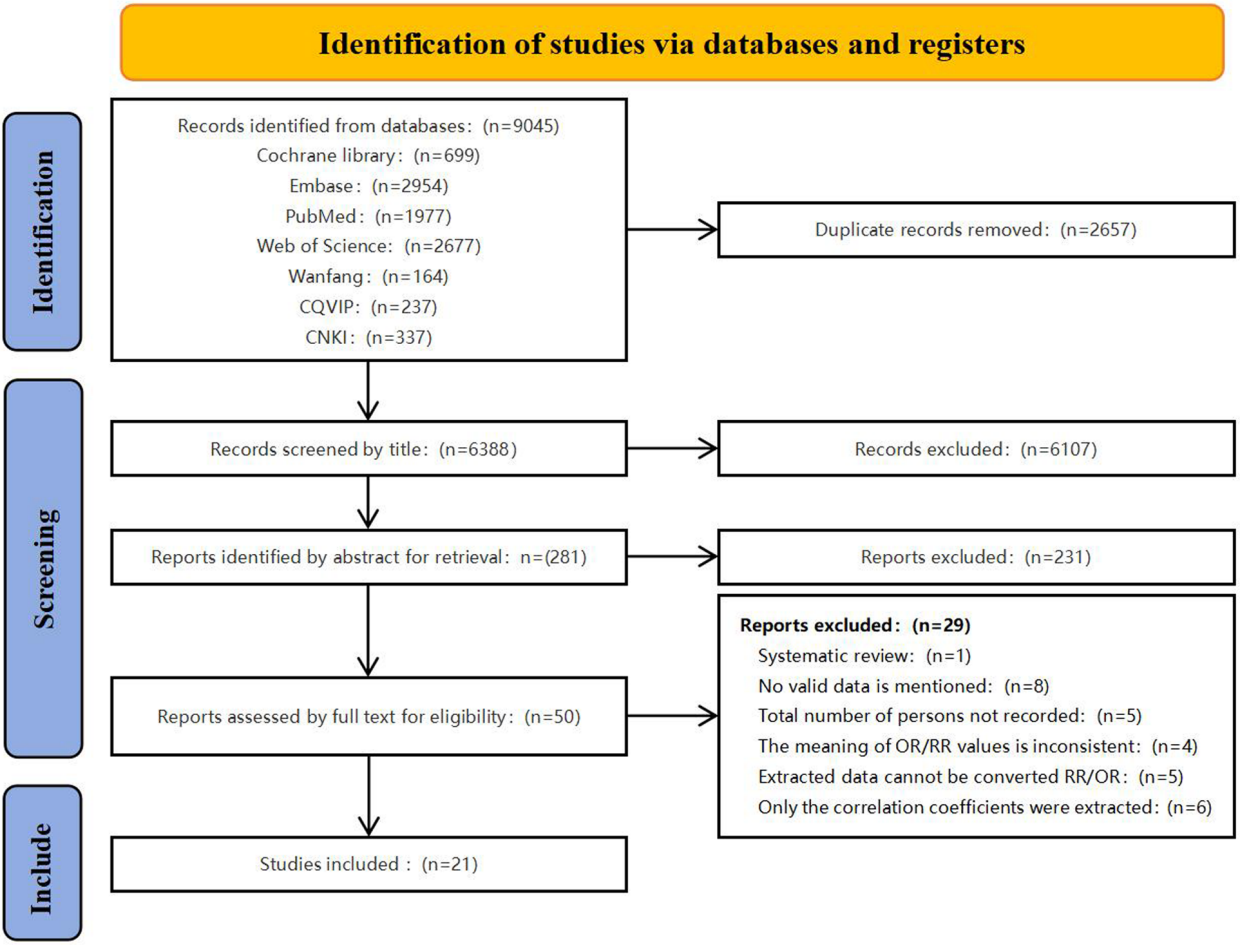
Flow chart for study selection in this systemic review and meta-analysis. CQVIP, China Science and Technology Journal Database; CNKI, China National Knowledge Infrastructure.

Figure 1 depicts the systematic literature search process. A total of 9,045 potentially eligible articles were obtained from diverse databases. Following the removal of 2,657 duplicate records, a comprehensive evaluation of titles and abstracts led to the identification of 50 relevant articles for full-text assessment. Among these, 29 articles were found to be incongruous with the inclusion criteria, as outlined in Supplementary Table S1. Ultimately, a total of 21 articles were included.

Table 1 shows the characteristics of the 21 included articles. Of the studies examining the effect of humidity on asthma, it is worth noting that two articles investigated the association between humidity and outpatient asthma consultations (13, 31), while five articles explored the relationship between humidity and Emergency Room Visits (ERVs) for asthma (32–36). Furthermore, five articles focused on the association between humidity and inpatient asthma (6, 37, 38). Four articles investigated the relationship between humidity and acute asthma attack (21, 39–41), and two articles provided the effect of humidity on asthma consultation and asthma control assessment, respectively, and its results demonstrated that relative humidity offered significant additional explanatory power to its model (42, 43). Among the studies on the effect of precipitation on asthma, two articles explored the relationship between precipitation and asthma emergency department visits (22, 33), three articles investigated the association of precipitation with asthma hospitalization (6, 37, 38), and three articles on the relationship between precipitation and asthma acute attack (16, 40, 41). Among the included studies, 12 were time-series studies, 4 were a case-crossover studies, 4 were cross-sectional studies, and 1 was ecological study. These studies were conducted in various countries, encompassing a total sample size of 1,052,960 participants.

**Table 1:** Basic characteristic of included studies.

First author	Year	Country	Study design	Gender	Age (years)	Sample size	Exposure factors	Outcomes	Quality
Longyan Li	2020	China	TS	M 13,874 F 16,380 N 1	≤14 15–64 ≥ 65	30,255	Relative humidity (lag7)	Asthma outpatient	4/5
Huayue Liu	2019	China	TS	M 1,684 F 2,249	18–64 ≥ 65	3,933	Relative humidity	Asthma hospitalization	3/5
Sutyajeet Soneja	2016	USA	C-C	M 45,226 F 70,695	≤4 5–17 18–64 ≥ 65	115,923	Precipitation	Asthma hospitalization	2/5
Marjan Kljakovic	1998	New Zealand	TS	NA	NA	3,844	Relative humidity	Consultations for asthma	2/5
Jae-Woo Kwon	2013	South Korea	C-C	NA	NA	656	Mean relative humidity	Visits to emergency room for asthma	3/5
Nana Mireku	2009	USA	TS	NA	1–18	25,401	Relative humidity	Children's asthma ED visits	4/5
Hehua Zhang	2020	China	TS	M 94,854 F 53,901	0–5 6–14 ≥ 15	173,747	Relative humidity	Asthma outpatient	4/5
Toshikazu Abe	2009	Japan	TS	M 52.8% F 47.2%	≤14 > 15	6,447	Maximum humidity Precipitation	Visits to emergency room for asthma	4/5
Mitsuo Hashimoto	2004	Japan	TS	NA	2–15	5,559	Average relative humidity	Visits to emergency room for asthma	4/5
Holly Ching-yu Lam	2016	China	TS	NA	<5 5–14 15–59 > 59	56,112	Relative humidity	Asthma hospitalization	4/5
Zahra Kanannejad	2023	Iran	TS	M 157 F 192	19–98	349	MAR, MAH, MARD	Asthma hospitalization	4/5
Zahra Kanannejad	2022	Iran	TS	M 144 F 67	≤18	211	MAR, MAH	Asthma hospitalization	4/5
Arun Kumar Sharma	2020	India	TS	M 25 F 36	18–35 35–49 ≥ 50	61	Precipitation Relative humidity	Acute attacks of asthma	3/5
Leah H. Schinasi	2020	USA	C-C	M 8,270 F 5,213	≤18	13,483	Precipitation	Acute attacks of asthma	4/5
Fei Li	2013	China	CS	NA	6–13	20,768	MAH	Asthma attacks	8/11
Selma Metintas	2010	Turkey	CS	NA	≥20	25,843	MAR, MAH	Asthma attacks	5/11
Jae-Woo Kwon	2016	South Korea	C-C	M 302 F 281	0–19 20–59 60–75 ≥ 75	660	Relative humidity	Visits to emergency room for asthma	4/5
Ju-Hyeong Park	2022	USA	TS	NA	0–18 19–65 ≥ 65	63,789	Precipitation	Visits to emergency room for asthma	4/5
S K Weiland	2004	Western Europe	ES	NA	6–7 13–14	463,801	MAH	Asthma attacks	8/10
David S. Kordit	2020	USA	CS	NA	0–18 ≥ 19	42,065	Relative humidity	Asthma hospitalization	8/11
Insung Kang	2023	USA	CS	M 14 F 39	NA	53	Relative humidity	Assessment of asthma control	7/11

Zahra Kanannejad's two articles in 2022 and 2023 have populations of children and adults, respectively. TS, time-series study; C-C, case-crossover analysis; CS, cross-sectional study; ES, ecological study; M, male; F, female; N, unknown; NA, not available; MAR, mean annual rainfall; MAH, mean annual humidity; MARD, mean annual rainy days.

### 3.2 Quality assessment

The majority of included studies can be classified as moderately high quality (total score ≥4). Supplementary Tables S2–4 provides a summary of these studies’ quality characteristics, with adjustments for confounding variables in the study design being the primary reason for the quality decline. When investigating the impact of humidity and precipitation on asthma risk, it is crucial to account for the effects of other meteorological factors and air pollutants. The quality of the studies were scored low if they failed to do so. Moreover, the cumulative effect of exposure over time on asthma occurrence was also a contributing factor to the quality scores.

### 3.3 Pooled and subgroups analysis

#### 3.3.1 Pooled analysis of the relationship between humidity and precipitation with asthma

**Figure 2:**
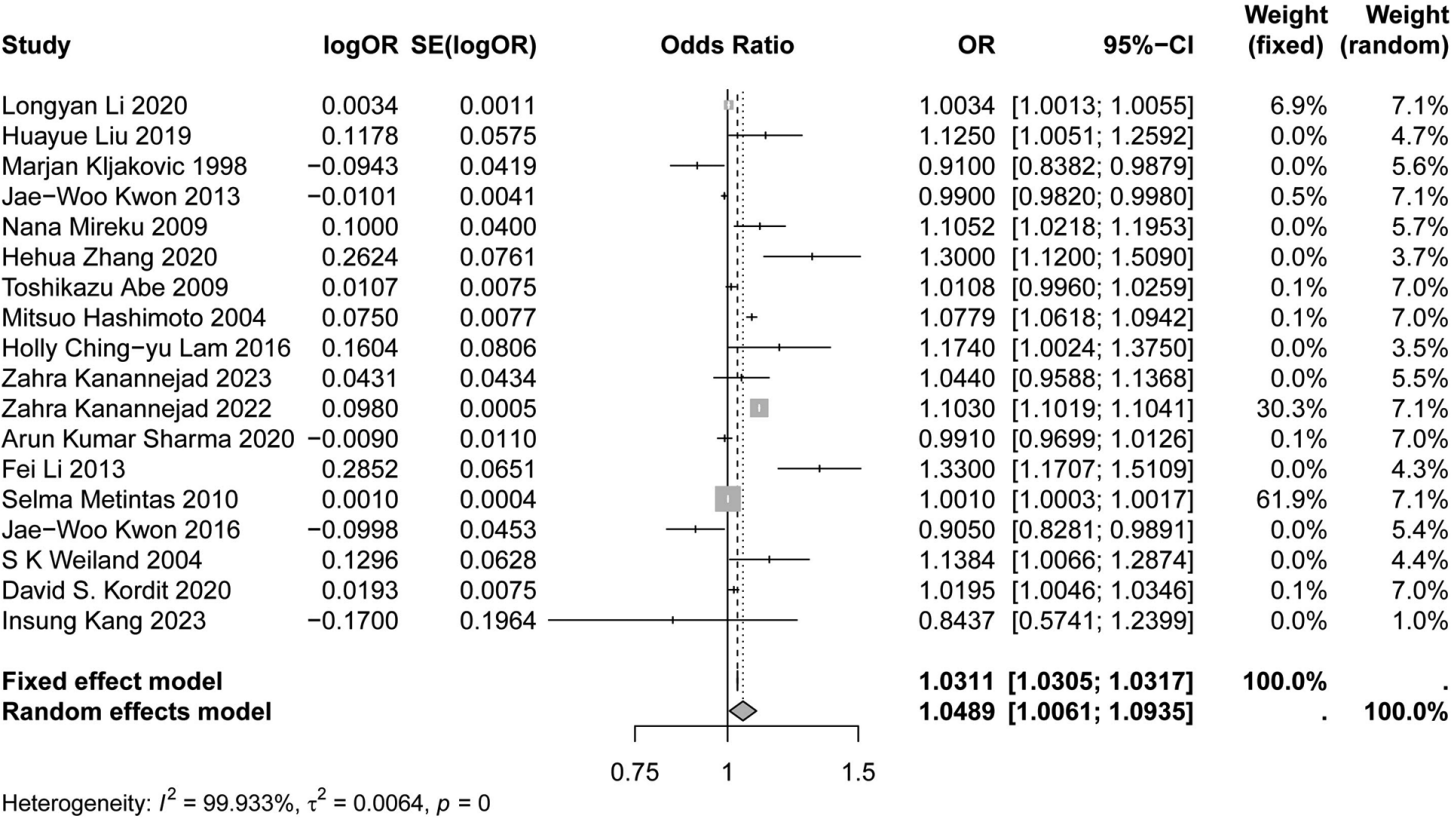
Forest plot of the relationship between humidity and asthma.

**Figure 3:**
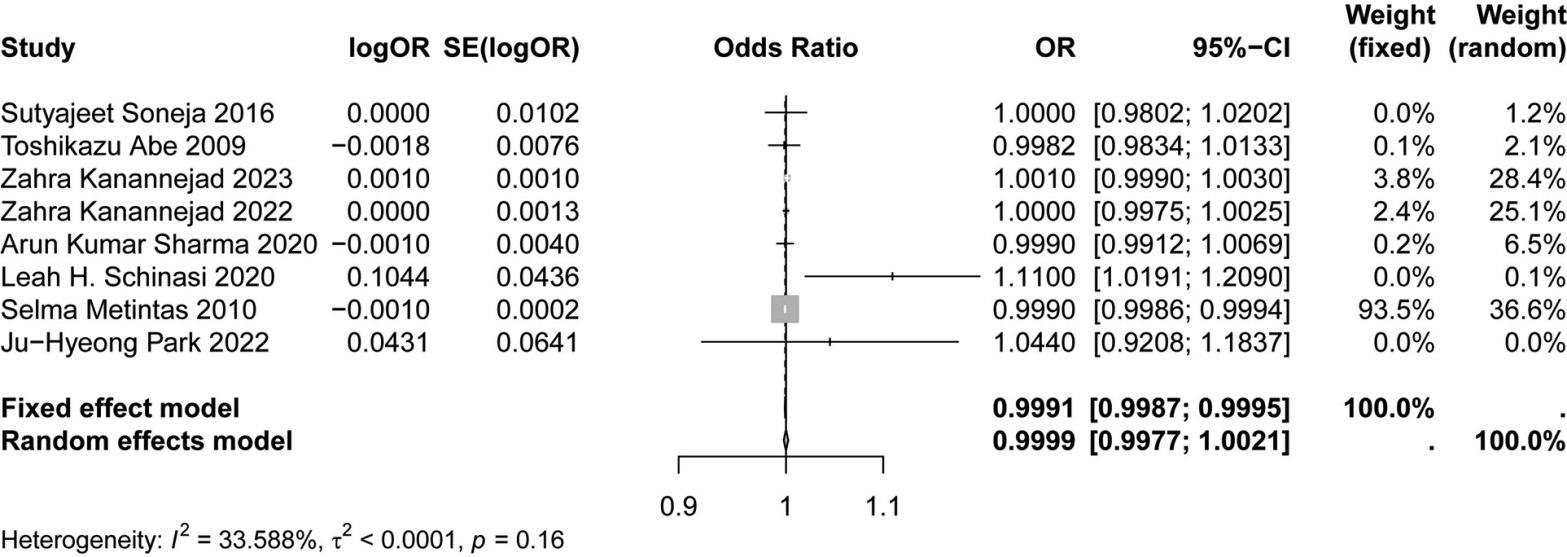
Forest plot of the relationship between precipitation and asthma.

Figures 2, 3 demonstrates the significant association between humidity, precipitation and asthma risk (OR: 1.0489; 95% CI: 1.0061, 1.0935; *p* < 0.05), (OR: 0.9991; 95% CI: 0.9987, 0.9995; *p* < 0.05). The results showed that, relative to the reference level, the incidence of asthma increased by 4.89% and decreased by 0.09% for each unit change in humidity and precipitation, respectively.

#### 3.3.2 Subgroup analyses of humidity and precipitation with asthma

Table 2 displays the results of three subgroup analyses, including age, regional development, and latitude.

**Table 2:** Subgroup analyses of association of asthma with humidity and precipitation.

Subgroup	Numbers of studies	Pooled OR (95% CI)	Heterogeneity	*P* of Egger's test
Humidity	18	**1.0489** (**1.0061; 1.0935)**	I^2^ = 99.933%, *P* < 0.001	0.9037
Age				
<18 years	8	**1.0898** (**1.0290; 1.1541)**	I^2^ = 99.892%, *P* < 0.001	0.6147
≥18 years	5	**1.0010** (**1.0003; 1.0017)**	I^2^ = 44.445%, *P* = 0.13	0.2730
Development level				
Developing countries or regions	9	**1.0927** (**1.0220; 1.1684)**	I^2^ = 99.968%, *P* < 0.001	0.8497
Developed countries or regions	9	1.0126 (0.9651; 1.0625)	I^2^ = 93.271%, *P* < 0.01	0.7507
Latitude level				
Low latitude	4	1.0600 (0.9921; 1.1325)	I^2^ = 96.897%, *P* < 0.01	0.4156
Middle latitude	8	1.0138 (0.9868; 1.0416)	I^2^ = 94.227%, *P* < 0.01	0.2982
High latitude	3	1.1298 (0.9502; 1.3433)	I^2^ = 66.149%, *P* = 0.05	0.8935
Precipitation	8	**0.9991 (0.9987; 0.9995)**	I^2^ = 33.588%, *P* = 0.16	0.0849
Age				
<18 years	4	1.0002 (0.9969; 1.0034)	I^2^ = 61.246%, *P* = 0.05	0.2852
≥18 years	5	**0.9991** (**0.9987; 0.9995)**	I^2^ = 12.918%, *P* = 0.33	0.5438
Development level				
Developing countries or regions	4	**0.9991** (**0.9987; 0.9995)**	I^2^ = 28.794%, *P* = 0.24	0.3127
Developed countries or regions	4	1.0013 (0.9895; 1.0132)	I^2^ = 51.607%, *P* = 0.10	0.1530
Latitude level				
Low latitude	3	1.0005 (0.9990; 1.0021)	I^2^ = 0%, *P* = 0.77	0.4967
Middle latitude	5	**0.9990** (**0.9986; 0.9994)**	I^2^ = 36.901%, *P* = 0.18	0.2632

Statistically significant results are in bold.

##### 3.3.2.1 Effect of humidity and precipitation on asthma by age

Table 2 indicates the results of subgroup analysis by age. In the <18 years group, for each unit rise in humidity, the rate of asthma risk increased by 8.98% (OR: 1.0898; 95%CI: 1.0290, 1.1541), while no significant effect of precipitation is observed (OR: 1.0002; 95%CI: 0.9969, 1.0034). In contrast, in the ≥18 years group, for each unit change in humidity, there was a 0.10% increase in the occurrence of asthma (OR: 1.0010; 95%CI: 1.0003, 1.0017), and an increase in precipitation leads to a slight 0.09% reduction in asthma risk (OR: 0.9991; 95%CI: 0.9987, 0.9995). (Supplementary Figures S1A, S2A).

##### 3.3.2.2 Effect of humidity and precipitation on asthma by regional development

Table 2 shows the results of subgroup analysis by regional development. In developing countries, both humidity and precipitation significantly impact asthma risk. Specifically, each unit increase in humidity results in a 9.27% increase in asthma risk (OR: 1.0927; 95% CI: 1.0220, 1.1684), while a unit increase in precipitation is linked to a 0.09% reduction in asthma risk (OR: 0.9991; 95% CI: 0.9987, 0.9995). In contrast, in developed countries, there is no significant association between asthma risk and either humidity (OR: 1.0126; 95% CI: 0.9651, 1.0625) or precipitation (OR: 1.0013; 95% CI: 0.9895, 1.0132), (details in Supplementary Figures S1B, S2B).

##### 3.3.2.3 Effect of humidity and precipitation on asthma by latitude

Table 2 reveals the results of subgroup analysis by latitude. Although the overall results did not reach statistical significance, the odds ratios (OR) for humidity in low, middle, and high latitudes were 1.0600 (95% CI: 0.9921, 1.1325), 1.0138 (95% CI: 0.9868, 1.0416), and 1.1298 (95% CI: 0.9502, 1.3433), respectively, with high latitudes showing the most pronounced effect on asthma risk due to humidity. For precipitation, middle latitudes demonstrated a protective effect, with each unit increase associated with a 0.10% reduction in asthma risk (OR: 0.9990; 95% CI: 0.9986, 0.9994). In contrast, low latitudes did not exhibit a significant association between precipitation and asthma risk (OR: 1.0005; 95% CI: 0.9990, 1.0021), (details in Supplementary Figures S1C, S2C).

### 3.4 Sensitivity analysis and publication bias

#### 3.4.1 Sensitivity analysis and publication bias in overall analyses

Sensitivity analyses have affirmed robust associations between both humidity and precipitation with asthma risk, with no significant publication bias detected in the overall analysis for either factor (Supplementary Figure S3, S4). The exclusion of one study slightly altered the precipitation-asthma association but did not undermine the overall robustness of the findings (40). Funnel plots in Supplementary Figures S5, S6 support the absence of publication bias.

#### 3.4.2 Sensitivity analyses and publication bias in subgroups

During sensitivity analyses examining the relationship between humidity and asthma, it was observed that the association is robust for adolescents under 18 years of age and across regions with varying levels of development. However, in adults over 18 years, the association becomes nonsignificant following the exclusion of the study by Selma Metintas. Additionally, the association between humidity and asthma varies across different latitudes after the exclusion of specific studies. In the sensitivity analysis of the relationship between precipitation and asthma, no change in the association was found for adolescents under 18 years of age and for developed regions upon the exclusion of any study. Nevertheless, in adults over 18 years, in developing regions, and in mid-latitude areas, the association between precipitation and asthma becomes nonsignificant after the exclusion of the study by Selma Metintas (Supplementary Figures S7, S8).

No publication bias was detected across all subgroups for both humidity and precipitation, as indicated by Egger's test results (*p* > 0.05). Supplementary Figures S9, S10 shows the corresponding funnel plots.

## 4 Discussion

### 4.1 The main findings

This systematic review and meta-analysis comprehensively assessed the association between humidity, precipitation, and asthma. The results indicate a heightened asthma risk with increased humidity, while higher precipitation levels appear to mitigate this risk. Sensitivity analyses further confirmed that the overall findings were robust.

### 4.2 Comparison of the results of subgroup analyses with similar studies

#### 4.2.1 The relationship between humidity and asthma

The pivotal role of humidity in the pathogenesis of asthma is chiefly ascribed to its influence on the epithelial barrier, potentially resulting in compromised mucus clearance and an elevated risk of allergic sensitization (44). A major consequence of altered humidity in the epithelial layer is the disruption of the epithelial barrier, leading to impaired mucus clearance by cilia (10). This disruption of mucosal integrity increases the risk of allergic sensitization and triggers the Th-2 inflammatory cascade, which plays a key role in asthma inflammation (45).

Experimental studies support that high humidity can promote the disruption of the airway epithelial mucosa integrity and trigger the Th-2 inflammatory cascade. For example, Duan's study (46) showed that exposure to high humidity or 0.5 mg/m^3^ formaldehyde alone had a small, insignificant effect on allergic asthma. However, simultaneous exposure to both significantly worsened pathological responses and airway hyper responsiveness. This indicates that environment with elevated humidity levels may intensify the detrimental impact of formaldehyde and other harmful agents on the airway epithelial barrier, consequently elevating the risk of allergen exposure. Furthermore, high humidity conditions may potentiate the activation of pulmonary transient receptor potential vanilloid 4 (TRPV4) ion channels, resulting in heightened airway inflammation and excessive mucus secretion, which can contribute to the pathogenesis of asthma (46).

Relevant studies indicate that high humidity levels exceeding 90% can induce oxidative stress, activate the TRPV1 pathway, and promote type I hypersensitivity, thereby contribute to the development and progression of asthma (47). Additionally, high humidity exposure may alter the structure and function of the gut microbiota, which can subsequently influence asthma development through the gut-lung axis (48). Moreover, Harun's study emphasized the role of grass pollen associated with humidity and rainfall during thunderstorms, causing early asthmatic responses in sensitized individuals (49).

#### 4.2.2 Relationship between humidity and asthma in young vs. adult individuals

After stratifying the data by age, among individuals younger than 18 years, we observed a stronger association between humidity and asthma risk in the asthmatic population. This finding aligns with current evidence suggesting that asthma is influenced by a combination of molecular damage that may occur during childhood and the less mature immune system of children (50). Furthermore, the respiratory system of children is still developing and more susceptible to environmental factors and the effects of humidity compared to adults (51, 52).

It is noteworthy that humidity levels can potentially clear harmful airway microorganisms by affecting the rheological properties of mucus and altering its osmotic pressure (10). This mechanism posits that optimal humidity levels, in contrast to excessive humidity, may contribute to a healthier airway environment by enhancing mucus clearance. This could be especially advantageous for individuals with asthma. Nevertheless, the efficacy of this mechanism may base on the individual's inherent mucus characteristics and overall health condition, including the integrity of the epithelial tissue. Additionally, Jonathan Thorsen's study highlighted a strong correlation between the oropharyngeal airway microbiota and age, which may contribute to the differential sensitivity to humidity among different age groups of asthmatic populations (53). This finding implies that age-related changes in the airway microbiome could influence how asthmatic individuals respond to humidity changes. For instance, younger individuals might exhibit different responses compared to older individuals due to variations in their microbiota composition. However, further study is needed to understand the interplay between age, microbiota, and humidity in asthma.

Regarding the attenuated association between humidity and asthma in adults, this may be due to the more mature airway development and stable airway microbiota compared to young individuals, making them less susceptible to the adverse effects of high humidity. Furthermore, adults are more likely to engage in proactive strategies to mitigate the impact of extreme weather conditions, such as refraining from outdoor activities during periods of high temperature and humidity (54).

#### 4.2.3 Asthmatic populations in developing countries or regions were more sensitive to humidity

When examining the subgroups based on regional development levels, we observed that although the heterogeneity remained unchanged in the developing region group, the combined OR increased. This indicates that individuals with asthma in developing countries were more sensitive to humidity. Cities in developing countries often struggle with higher levels of air pollution compared to cities in developed countries. These pollutants can carry harmful and allergenic substances that interact with humidity, thereby increasing the risk of asthma attacks (55).

Bryant-Stephens’ study demonstrated that healthy housing is also an important social determinant of asthma, and that housing conditions in economically underdeveloped areas are often substandard (56). Moreover, Biagtan's study suggests that indoor environments with high humidity and inadequate ventilation promote mold and mite growth, which exacerbates asthma symptoms (57). Furthermore, there are differences in the development and functioning of the immune system between developing and developed countries. Asthmatics in developing countries may have a higher risk of infection, which over activates the immune system and leads to increased sensitivity to humidity (58).

#### 4.2.4 Asthmatic populations at high latitudes were more sensitive to humidity

Subgroup analyses by latitude levels reduced heterogeneity, and sensitivity analyses confirmed consistent results. While statistical significance was not evident in low, middle, and high latitude subgroups, this may be attributed to the smaller sample sizes in these subgroups. Higher latitudes have larger OR values than low and middle latitudes, showing that humidity variation has a greater impact on asthma risk at higher latitudes, consistent with previous research.

Krstic's research demonstrated that every 10° increase in latitude was associated with a 2% increase in the prevalence of adult asthma (*p* < 0.001) (59). Yu's study suggests that the relationship between latitude and allergic diseases may be influenced by vitamin D and sunlight exposure, which play a role in modifying the risk of allergic reactions (60). Based on these findings, in high-latitude regions, diminished levels of vitamin D, attributable to decreased sunlight exposure and ultraviolet intensity, may adversely impact immune function. Consequently, this may increase the body's susceptibility to asthma triggers associated with risk factors prevalent in high-humidity environment.

#### 4.2.5 The relationship between precipitation and asthma

Our findings suggest that precipitation exerts a protective influence on asthma, potentially attributable to its capacity to efficiently remove particulates and allergens from the atmosphere. This process may consequently diminish exposure to risk factors for individuals with asthma (17). However, Soneja et al. have shown that summer extreme precipitation events increase asthma risk of hospitalization by 11% (OR: 1.11; 95% CI: 1.06, 1.17). Moreover, the risk of asthma attributable to extreme precipitation events varied across age groups (37). Nassikas et al. found that large-scale short-term precipitation events may induce increased Fractional Exhaled Nitric Oxide (FeNO) and airway inflammation in adolescents, especially asthmatics (61). This may be due to an increase in air humidity after heavy precipitation, which induces asthma (62). Furthermore, heavy rainfall may impede the ability of some patients with mild asthma to travel to the hospital, resulting in a decrease in asthma-related hospital visits (63, 64). Moderate precipitation may help reduce air pollutants, while extreme precipitation in summer may increase the concentration of allergens such as mold due to high temperature and humidity, thereby increasing airway reactivity and triggering asthma attacks, especially for children whose respiratory systems are not fully mature.

#### 4.2.6 Relationship between precipitation and asthma in young vs. adult individuals

After stratification by age, precipitation was a protective factor and heterogeneity was reduced among those aged older than 18 years. However, adolescents under 18 years were not sensitive to the protective effect of precipitation, possibly because participants under 18 years are more sensitive to the effect of relative humidity, which offsets the protective effect of precipitation. Some researchers found that precipitation was inversely associated with the risk of asthma hospitalizations, exacerbations, and prevalence of asthma in people over 18 years of age (37, 40, 41). This may be due to the range of protective measures that adults take against extreme weather (54).

#### 4.2.7 Relationship between precipitation and asthma in developing countries or regions

In the subgroup of regional development level, the heterogeneity of the developing area decreased, and the protective effect of precipitation on asthmatics was more pronounced in developing area. Because cities in developing countries generally face higher air pollution problems than cities in developed countries, the cleaning effect of precipitation on air pollutants in developing regions is stronger than that in developed countries (65). In addition, traffic conditions in economically underdeveloped areas may be poor, and heavy precipitation may hinder asthma patients from seeking medical care (63, 64).

#### 4.2.8 Relationship between precipitation and asthma in middle latitudes

Subgroup analysis revealed that the sensitivity of latitude was relatively stable. The protective effect of precipitation on asthma was more pronounced in middle latitudes. This may be because the extreme temperatures in high and low latitudes affect the protective effect of precipitation on asthma, while in middle latitudes, this effect is less pronounced (66). Additionally, the long-term high temperature and humidity environment in low latitudes may weaken the protective effect of precipitation on asthma due to the overgrowth of allergens such as mold (67). Therefore, the impact of precipitation on asthma is more significant in middle latitudes.

### 4.3 Possible links of humidity and precipitation to asthma

On the one hand, we observed that increased relative humidity was associated with a rise in the risk of asthma. This may be due to the increased moisture in the air under high humidity conditions, which provides a breeding environment for allergens such as dust mites and mold spores, thus increasing the possibility of asthma attacks (49). On the other hand, increased precipitation appeared to be associated with a reduced risk of asthma. Precipitation increases the amount of water in the air, but it also has the effect of cleaning the air by washing away pollen, allergens, and pollutants, thereby reducing respiratory stimuli. While precipitation may increase relative humidity in the air, its cleansing effect could potentially mitigate the negative impacts of high humidity-induced elevated allergen concentrations in the short term. Rainwater can flush away pollen, allergens, and pollutants from the atmosphere, thereby reducing respiratory irritants and lowering the risk of asthma (17). Furthermore, the temporal effects of precipitation and relative humidity on asthma risk exhibit differences. Short-term precipitation may have a positive impact on asthma risk, but after the rain ceases or with sustained heavy precipitation, the increase in relative humidity could heighten the risk of asthma.

At the same time, asthma risk is associated with a variety of environmental factors, and individuals may respond differently to these factors (45, 68). Therefore, more in-depth research are needed in the future to consider the incidence of asthma in different regions and under different climatic conditions, as well as the health status and environmental exposure history of individuals. This will help us to more fully understand the relationship between environmental factors and asthma and provide more effective strategies for the prevention and treatment of asthma.

### 4.4 Strengths and limitations

This review has several strengths. It is the first review to investigate the relationship between humidity, precipitation, and the risk of asthma attacks. The inclusion of a large sample size with controlled socioeconomic and demographic factors enhanced the study's robustness and generalizability. Asthma-related risks were examined using a variety of epidemiological research methods, providing strong evidence for the risks associated with asthma and the causality between humidity and precipitation changes.

This review faces limitations. High or moderate heterogeneity in meta-analyses suggests that unmeasured factors like air pollution and pollen may affect results, which were excluded due to data unavailability. Gender's role in asthma was unexplored due to limited studies, potentially biasing interpretations. The lack of direct data on humidity-precipitation interactions and varying reference levels for asthma risk complicates the analysis, highlighting the need for future meta-analyses to address these issues.

## 5 Conclusions

In summary, this meta-analysis provides robust evidence of a significant relationship of humidity and precipitation with asthma. For humidity, age <18 years, developing regions, and high latitudes appear to be associated with higher risk of asthma. For precipitation, the association with reduced asthma risk was more pronounced in individuals age ≥18 years old, developing regions, and the middle latitudes. These findings not only enhance our understanding of the adverse impact of humidity and precipitation fluctuations on respiratory health but also highlight the need for targeted interventions to mitigate the risk of asthma for these vulnerable groups. Future research should investigate the mechanistic underpinnings of how humidity and precipitation affects asthma attacks, explore the possible interactions between humidity and precipitation, and develop strategies to adapt to these environmental factors.

## Data Availability

The raw data supporting the conclusions of this article will be made available by the authors, without undue reservation.

## Supplementary material

Supplementary Figure S1 Subgroup analysis of humidity and asthma.

Supplementary Figure S2 Subgroup analysis of precipitation and asthma.

Supplementary Figure S3 Sensitivity analysis of humidity and asthma.

Supplementary Figure S4 Sensitivity analysis of precipitation and asthma.

Supplementary Figure S5 Humidity and asthma funnel plot.

Supplementary Figure S6 Precipitation and asthma funnel plot.

Supplementary Figure S7 Sensitivity analysis of the relationship between asthma and humidity in different subgroups; A. Age; B. Development level; C. Latitude interval.

Supplementary Figure S8 Sensitivity analysis of the relationship between asthma and precipitation in different subgroups; A. Age; B. Development level; C. Latitude interval.

Supplementary Figure S9 Funnel plot of the relationship between asthma and humidity in different subgroups; A. Age; B. Development level; C. Latitude interval.

Supplementary Figure S10 Funnel plot of the relationship between asthma and precipitation in different subgroups; A. Age; B. Development level; C. Latitude interval.
