# Supplementary figures and images for: Association of humidity and precipitation with asthma: a systematic review and meta-analysis
Source: Front Allergy. 2024 Dec 6;5:1483430. doi: 10.3389/falgy.2024.1483430 (PMC11659254; doi:10.3389/falgy.2024.1483430)

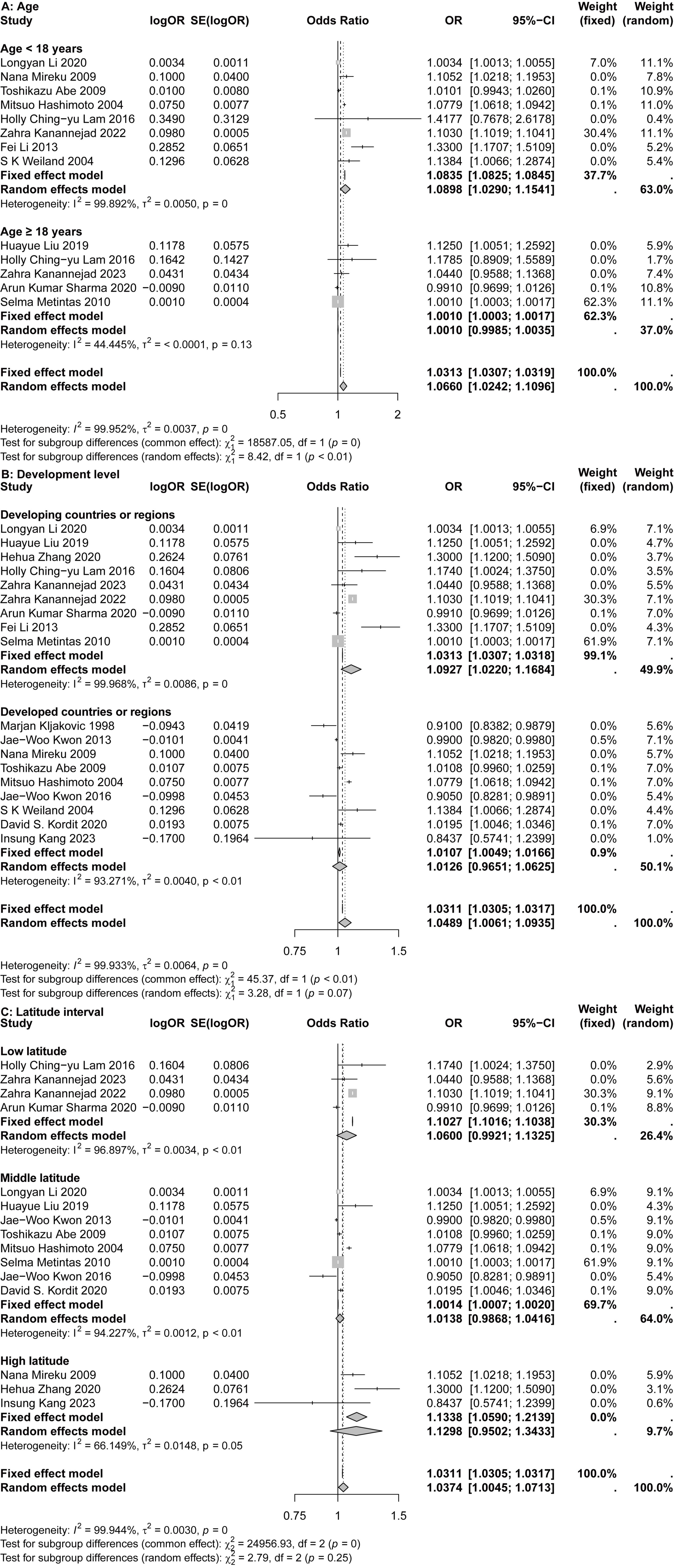

Supplement: Supplementary Figure S1 — Subgroup analysis of humidity and asthma. [file Image1.jpeg]

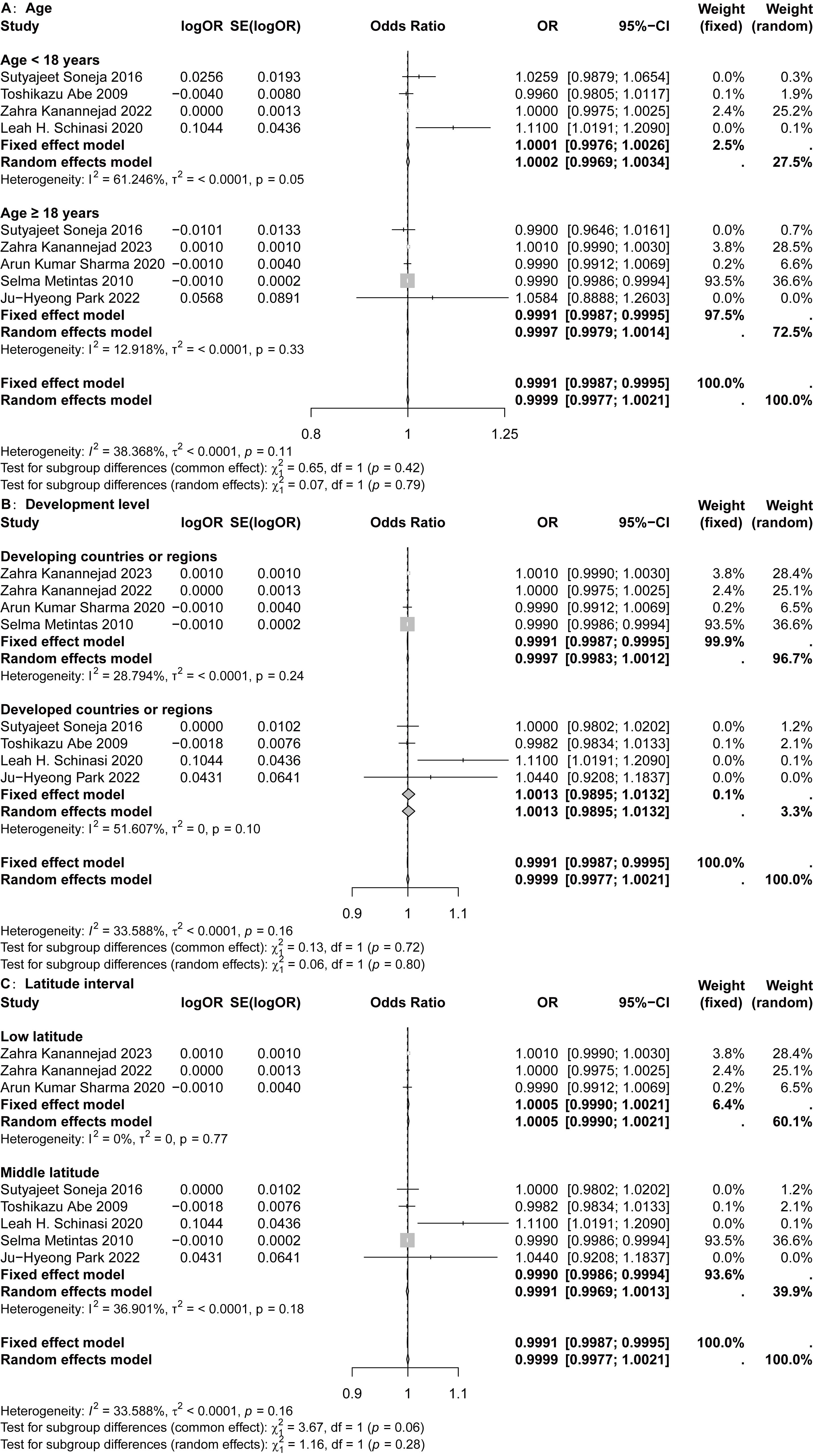

Supplement: Supplementary Figure S2 — Subgroup analysis of precipitation and asthma. [file Image2.jpeg]

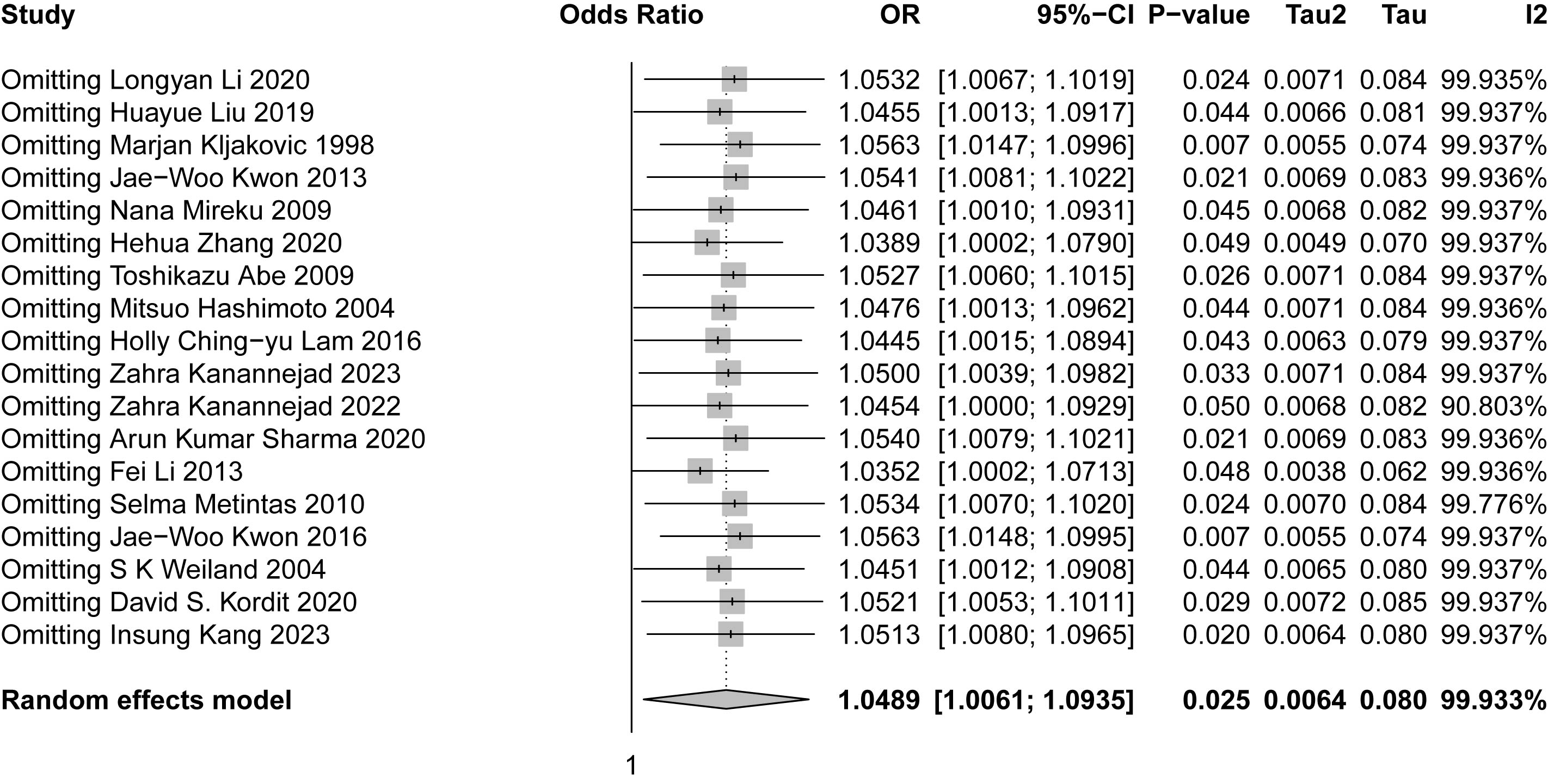

Supplement: Supplementary Figure S3 — Sensitivity analysis of humidity and asthma. [file Image3.jpeg]

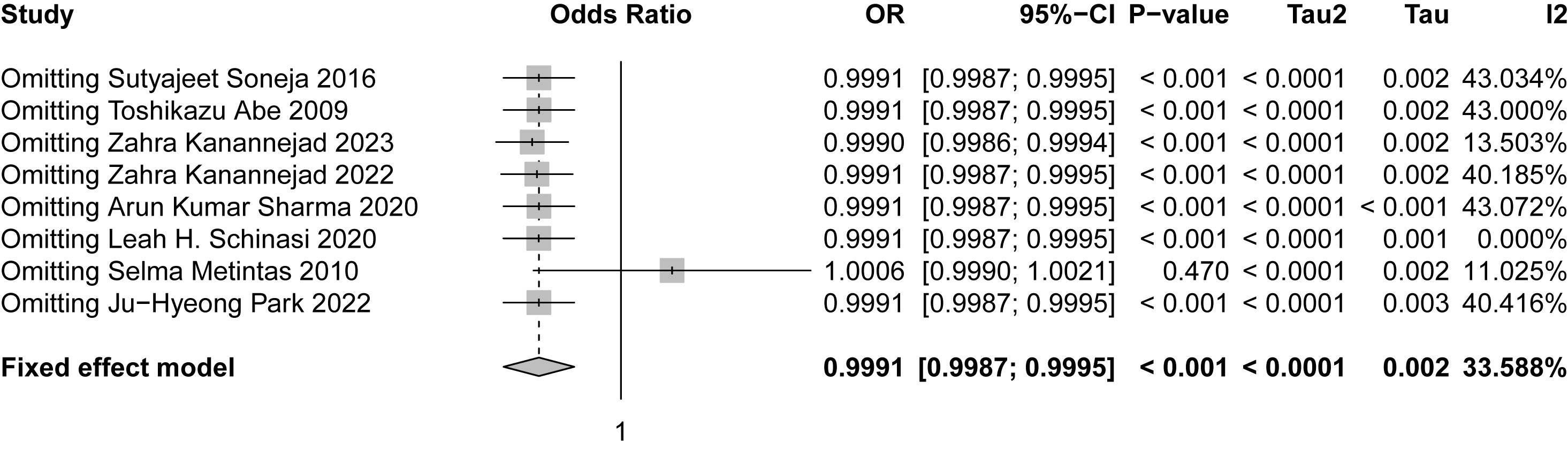

Supplement: Supplementary Figure S4 — Sensitivity analysis of precipitation and asthma. [file Image4.jpeg]

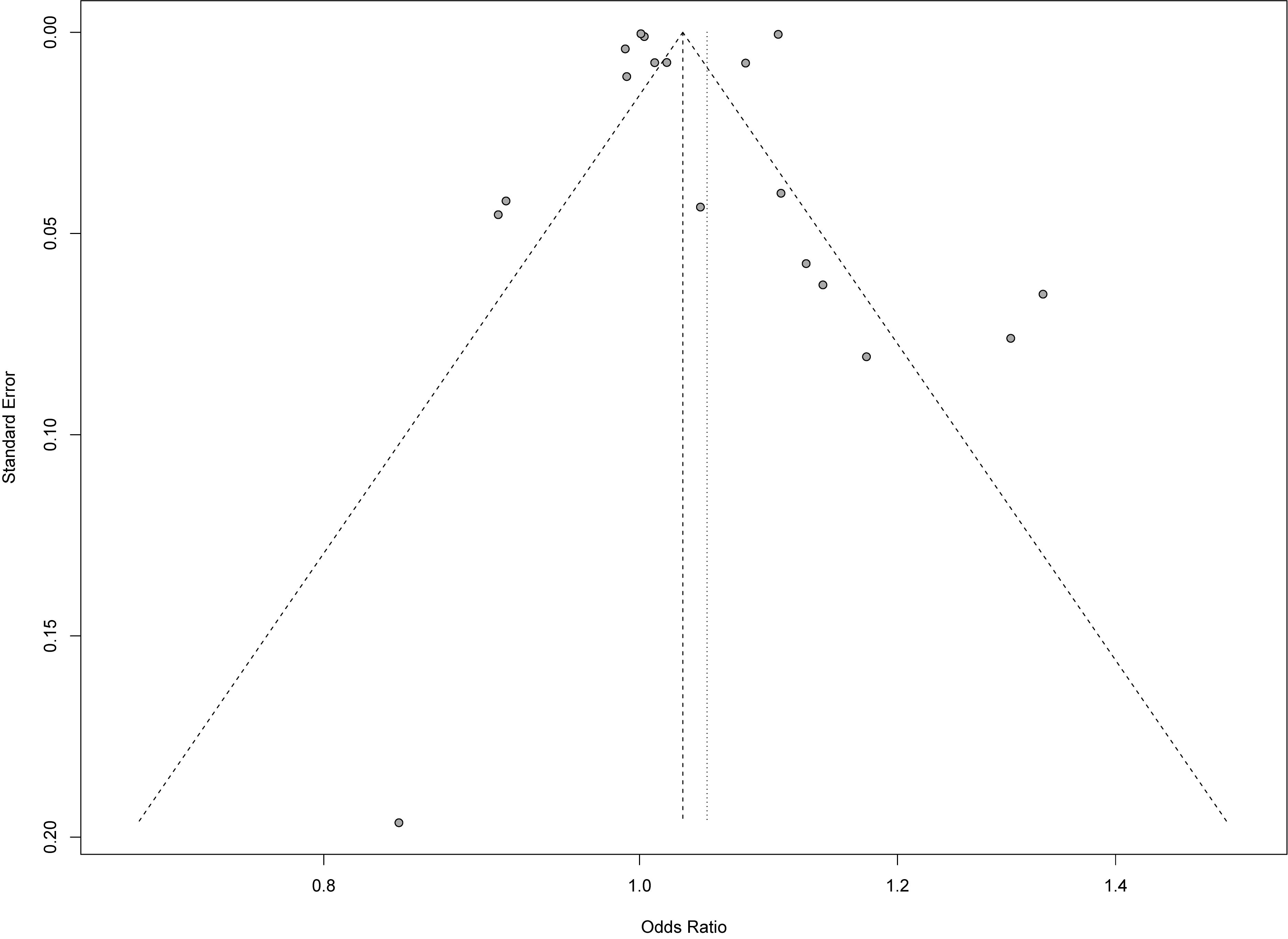

Supplement: Supplementary Figure S5 — Humidity and asthma funnel plot. [file Image5.jpeg]

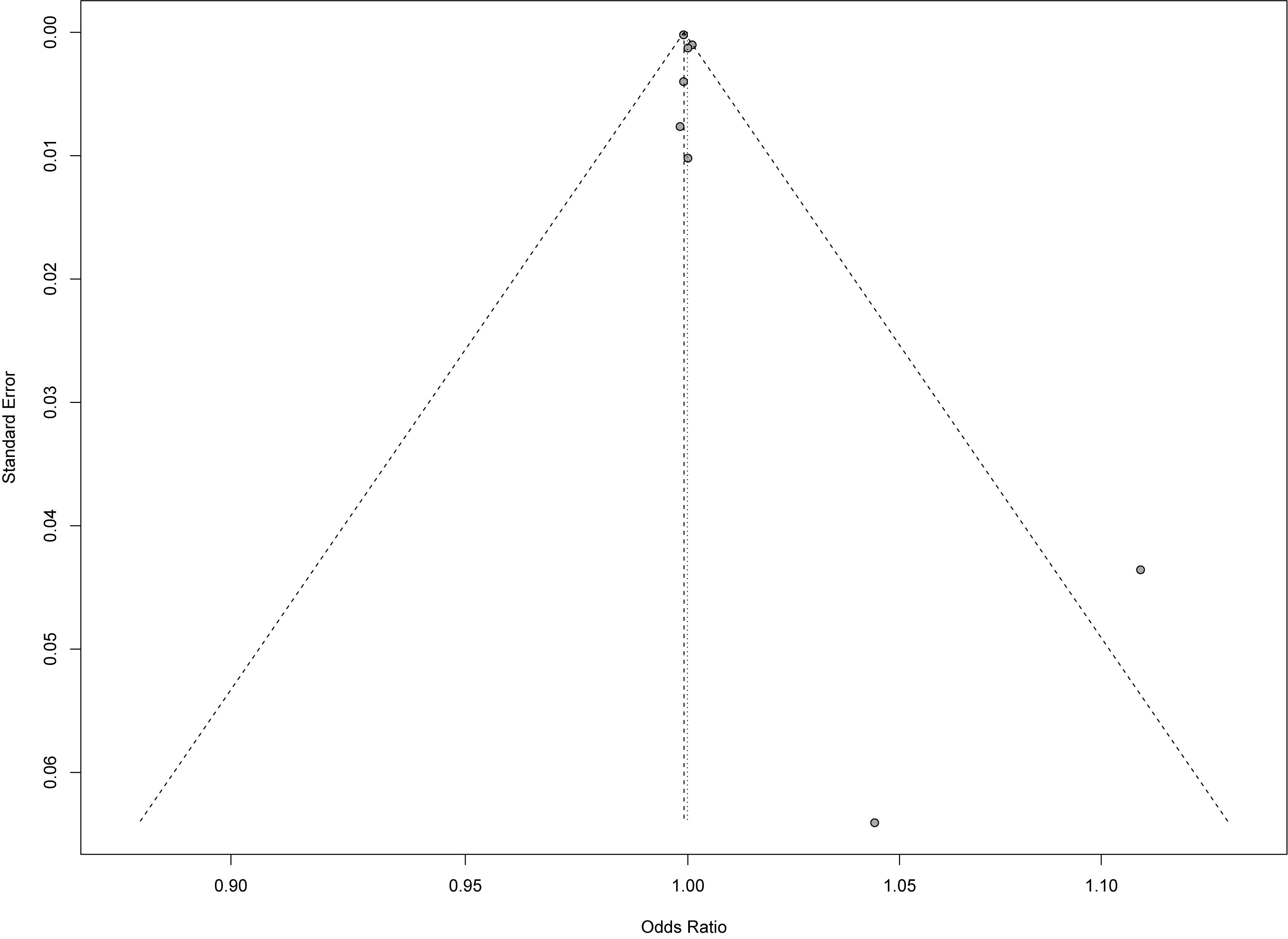

Supplement: Supplementary Figure S6 — Precipitation and asthma funnel plot. [file Image6.jpeg]

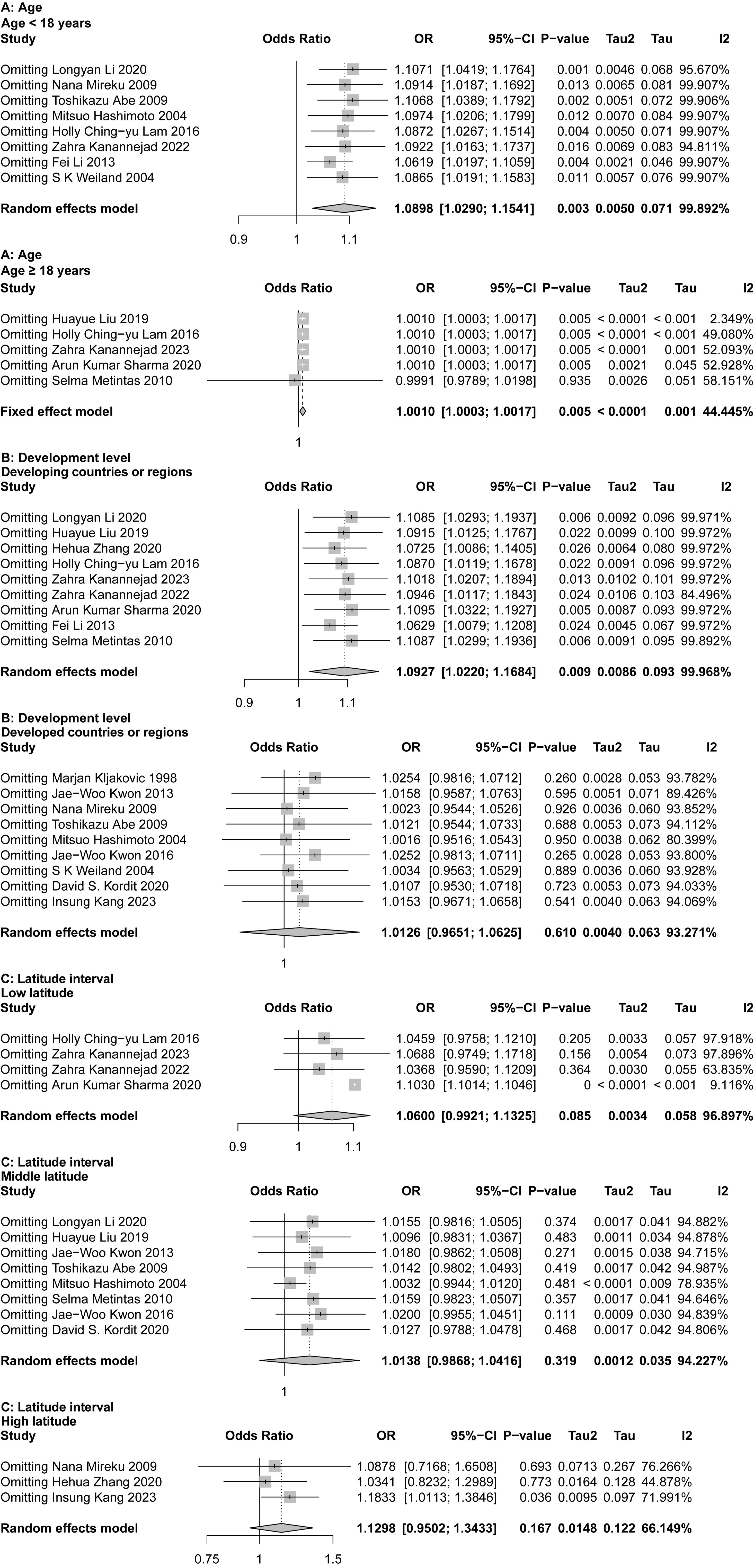

Supplement: Supplementary Figure S7 — Sensitivity analysis of the relationship between asthma and humidity in different subgroups; A. Age; B. Development level; C. Latitude interval. [file Image7.jpeg]

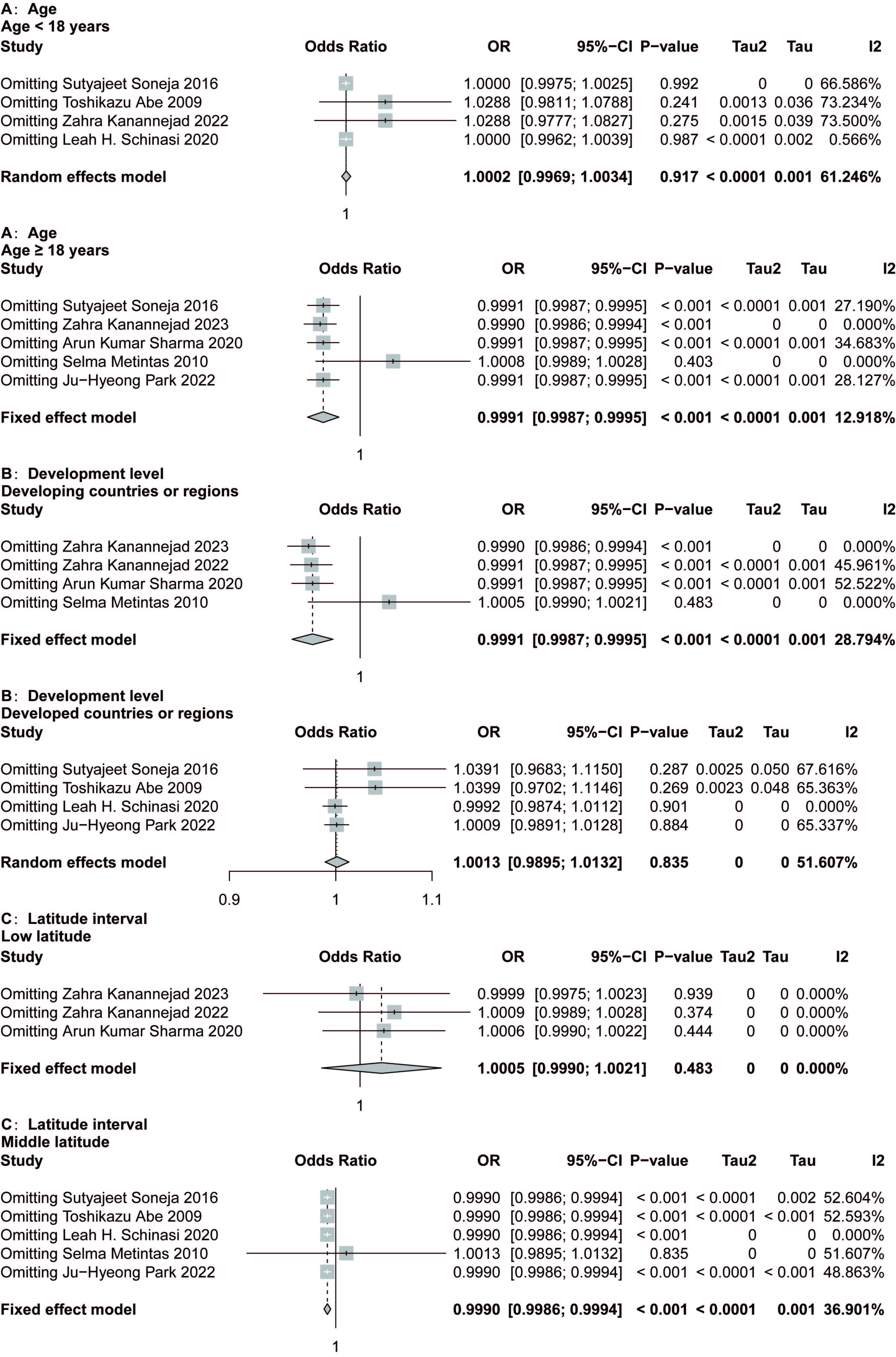

Supplement: Supplementary Figure S8 — Sensitivity analysis of the relationship between asthma and precipitation in different subgroups; A. Age; B. Development level; C. Latitude interval. [file Image8.jpeg]
