# Supplementary material for: Association of humidity and precipitation with asthma: a systematic review and meta-analysis
Source: Front Allergy. 2024 Dec 6;5:1483430. doi: 10.3389/falgy.2024.1483430 (PMC11659254; doi:10.3389/falgy.2024.1483430)
Supplement: Supplementary file 11 [file Table1.docx]

**TABLE S1 Characteristics of 29 excluded studies**

| First Author | Year | Country | Study Design | Gender | Age (Years) | Sample Size | Exposure Factors | Outcome | Reasons for exclusion |
| --- | --- | --- | --- | --- | --- | --- | --- | --- | --- |
| Chan Lu | 2023 | China | C | M&F | 3-6 | 8689 | Daily mean Relative Humidity | Not mentioned | Ⅲ |
| Huimin Zhao | 2022 | China | TS | M&F | 3-12 | 1108 | Mean monthly Humidity | Asthma outpatient | Ⅴ |
| Xiaomei Liu | 2007 | China | TS | M&F | < 14 | 445 | Mean monthly humidity | Asthma hospitalization | Ⅴ |
| 0 V J Rossi | 1993 | Finland | TS | M&F | 15-85 | 232 | Humidity  Rainfall | Visits to emergency room for asthma | Ⅴ |
| T. O. Lim | 1991 | Malaysia | CS | NA | NA | 2106 | Daily Relative Humidity Daily Rainfall | Visits to emergency room for asthma | Ⅴ |
| Anna Romaszko-Wojtowicz | 2020 | Poland | TS | NA | All ages | 1449 | Relative Humidity Rainfall sum | Asthma hospitalization | Ⅳ |
| N Adamia | 2012 | Georgia | CS | M&F | 5–16 | 4559 | Not mentioned | Not mentioned | Ⅵ |
| Yu, H. R | 2019 | China | C-C | M&F | All ages | NA | Not mentioned | Visits to emergency room for asthma | Ⅱ |
| Panagiotis t.nastos | 2008 | Greece | TS | NA | 0–4 5–14 | NA | Relative Humidity Absolute Humidity | Asthma hospitalization | Ⅱ |
| M.A.Ivey | 2003 | Trinidad | TS | M&F | All ages | 45842 | Total Rainfall Relative Humidity | Visits to emergency room for asthma | Ⅳ |
| Muge Akpinar-Elci | 2015 | Grenada | TS | M&F | 20.0±1.80 | 4411 | Rainfall Relative Humidity | Visits to emergency room for asthma | Ⅴ |
| Marsha A Ivey | 2001 | Trinidad | TS | M&F&N | All ages | 27848 | Rainfall Relative Humidity | Visits to emergency room for asthma | Ⅳ |
| Yabin Hu | 2020 | China | TS | M&F | 0–17 | 23103 | Relative Humidity | Visits to emergency room for asthma | Ⅲ |
| Kostas N. Priftis | 2006 | Greece | TS | NA | 0–14 | 25412 | Relative Humidity Precipitation | Asthma hospitalization | Ⅴ |
| Wei Han | 2022 | China | ES | M&F | ≥ 18 | 10549 | Relative Humidity | Asthma hospitalization | Ⅴ |
| Alberto Arnedo-Pena | 2013 | Western Europe | ES | NA | 6–7 13–14 | NA | Relative Humidity  Rainfall | Asthma in schoolchildren | Ⅱ |
| Mónica Rodrigues | 2021 | Portugal | TS | M&F | 0–14 | NA | Relative Humidity  Rainfall | Asthma hospitalization | Ⅱ |
| Rubing Pan | 2019 | China | TS | M&F | 0–18 | 32238 | Humidex | Asthma hospitalization | Ⅵ |
| AKIRA EHARA | 2000 | Japan | CC | M&F | 0.1–16.6 | 205 | Low Humidity | Asthmatic symptoms patients | Ⅵ |
| Vakhtang Beridze | 2018 | Georgia | CS | NA | 5-17 | 5319 | Not mentioned | Not mentioned | Ⅵ |
| Miami Kadhim Yousif | 2019 | Iraq | TS | M&F | 1-5 6-13 | 1043 | Relative Humidity | Asthma outpatient | Ⅴ |
| Andrew Rorie | 2021 | USA | RE | NA | NA | NA | Not mentioned | Not mentioned | Ⅰ |
| Mark P.C. Cherrie | 2021 | UK | ES | NA | NA | NA | Precipitation Relative Humidity | Asthma hospitalization | Ⅱ |
| Abdallah A. Abba | 2021 | Nigeria | CS | M&F | ≥ 16 | 87 | Humidity | Asthma outpatient | Ⅴ |
| J P Buckley | 2010 | USA | C-C | NA | > 18 | 53156 | Temperatures | Visits to emergency room for asthma | Ⅲ |
| Arjita Rai | 2023 | USA | C-C | M&F | All ages | 970903 | Precipitation | Visits to emergency room for asthma | Ⅵ |
| A Loftus | 2014 | Ireland | ES | NA | 1–14 | NA | Relative Humidity | Asthma hospitalization | Ⅵ |
| D. Hervás | 2015 | Spain | ES | NA | 5-14 | 371 | Relative Humidity | Visits to emergency room for asthma | Ⅳ |
| Kirthana U. Kunikullaya | 2017 | India | TS | NA | ≥ 18 | NA | Relative Humidity  Rainfall | Asthma hospitalization | Ⅵ |

C: Cohort study; TS: Time-series study; CS: Cross-sectional study; C-C: Case-crossover analysis; ES: Ecological study; CC: Case-control study; RE: Review.

M: Male; F: Female; NA: Not available; N: Unknown.

The reasons for exclusion: I: Systematic review. II: Total number of persons not recorded. III: The meaning of OR/RR values is inconsistent. IV: Extracted data cannot be converted RR/OR. Ⅴ: Only the correlation coefficients were extracted. Ⅵ: No valid data is mentioned.
