# Supplementary material for: Association of humidity and precipitation with asthma: a systematic review and meta-analysis
Source: Front Allergy. 2024 Dec 6;5:1483430. doi: 10.3389/falgy.2024.1483430 (PMC11659254; doi:10.3389/falgy.2024.1483430)
Supplement: Supplementary file 12 [file Table2.docx]

**TABLE S2 Quality assessment of time-series analysis and case-crossover studies**

| First Author | Year | Asthma diagnosis (0 to 1 point) | Quality of humidity/rainfall exposure level  (0 to 1 point) | Adjustment for confounders  (0 to 3 point) | Total score |
| --- | --- | --- | --- | --- | --- |
| Longyan Li | 2020 | 1 | 1 | 2 | 4/5 |
| Huayue Liu | 2019 | 1 | 1 | 1 | 3/5 |
| Sutyajeet Soneja | 2016 | 1 | 1 | 0 | 2/5 |
| Marjan Kljakovic | 1998 | 0 | 1 | 1 | 2/5 |
| Jae-Woo Kwon | 2013 | 1 | 1 | 1 | 3/5 |
| Nana Mireku | 2009 | 1 | 1 | 2 | 4/5 |
| Hehua Zhang | 2020 | 1 | 1 | 2 | 4/5 |
| Toshikazu Abe | 2009 | 1 | 1 | 2 | 4/5 |
| Mitsuo Hashimoto | 2004 | 1 | 1 | 2 | 4/5 |
| Holly Ching-yu Lam | 2016 | 1 | 1 | 2 | 4/5 |
| Zahra Kanannejad | 2023 | 1 | 1 | 2 | 4/5 |
| Zahra Kanannejad | 2022 | 1 | 1 | 2 | 4/5 |
| Arun Kumar Sharma | 2020 | 1 | 1 | 1 | 3/5 |
| Leah H. Schinasi | 2020 | 1 | 1 | 2 | 4/5 |
| Jae-Woo Kwon | 2016 | 1 | 1 | 2 | 4/5 |
| Ju-Hyeong Park | 2022 | 1 | 1 | 2 | 4/5 |

Since no validated scales of time-series and case-crossover studies were recommended by New Castle Ottawa and Cochrane risk of bias tool, we evaluated the validity based on Mustafic’s study . Three components were assessed, including asthma diagnosis (0 to 1 point),Quality of humidity/rainfall exposure level (0 to 1) and adjustment for confounders (0 to 3).
