# Supplementary material for: Association of humidity and precipitation with asthma: a systematic review and meta-analysis
Source: Front Allergy. 2024 Dec 6;5:1483430. doi: 10.3389/falgy.2024.1483430 (PMC11659254; doi:10.3389/falgy.2024.1483430)
Supplement: Supplementary file 13 [file Table3.docx]

**TABLE S3 Quality assessment of ecological studies**

| First Author | Year | Representativeness of the Sample  (0 to 1 point) | Sample Size  (0 to 1 point) | Non-Respondents (Max. 1) | Ascertainment of Exposure  (0 to 2 point) | Comparable Subjects in Different Outcome Groups. Confounding Factors Controlled  (0 to 2 point) | Assessment of Outcome  (0 to 2 point) | Statistical Test (0 to 1 point) | Total score |
| --- | --- | --- | --- | --- | --- | --- | --- | --- | --- |
| S K Weiland | 2004 | 1 | 1 | 0 | 2 | 2 | 1 | 1 | 8/10 |

The study was ecological and assessed using the NOS scale
