# Supplementary material for: Association of humidity and precipitation with asthma: a systematic review and meta-analysis
Source: Front Allergy. 2024 Dec 6;5:1483430. doi: 10.3389/falgy.2024.1483430 (PMC11659254; doi:10.3389/falgy.2024.1483430)
Supplement: Supplementary file 14 [file Table4.docx]

**TABLE S4 Quality assessment of cross-sectional studies**

| First Author | Year | Define the source of information (survey, record review) | List inclusion and exclusion criteria for exposed and unexposed subjects (cases and controls) or refer to previous publications | Indicate time period used for identifying patients | Indicate whether or not subjects were consecutive if not population-based | Indicate if evaluators of subjective components of study were masked to other aspects of the status of the participants | Describe any assessments undertaken for quality assurance purposes (e.g., test/retest of primary outcome measurements) | Explain any patient exclusions from analysis | Describe how confounding was assessed and/or controlled | If applicable, explain how missing data were handled in the analysis | Summarize patient response rates and completeness of data collection | Clarify what follow-up, if any, was expected and the percentage of patients for which incomplete data or follow-up was obtained | Total score |
| --- | --- | --- | --- | --- | --- | --- | --- | --- | --- | --- | --- | --- | --- |
| Fei Li | 2013 | 1 | 1 | 1 | 0 | 1 | 1 | 1 | 1 | 0 | 1 | 0 | 8/11 |
| Selma Metintas | 2010 | 1 | 0 | 1 | 0 | 0 | 1 | 0 | 1 | 0 | 1 | 0 | 5/11 |
| David S. Kordit | 2020 | 1 | 0 | 1 | 1 | 1 | 1 | 0 | 1 | 1 | 1 | 0 | 8/11 |
| Insung Kang | 2023 | 1 | 0 | 1 | 1 | 0 | 1 | 0 | 1 | 1 | 1 | 0 | 7/11 |

The above studies were cross-sectional and assessed using AHRQ scale
